# Molecular cloning, bioinformatics analysis, and expression of small heat shock protein beta-1 from *Camelus dromedarius*, Arabian camel

**DOI:** 10.1371/journal.pone.0189905

**Published:** 2017-12-29

**Authors:** Manee M. Manee, Sultan N. Alharbi, Abdulmalek T. Algarni, Waleed M. Alghamdi, Musaad A. Altammami, Mohammad N. Alkhrayef, Basel M. Alnafjan

**Affiliations:** 1 National Center for Genomic Technology, King Abdulaziz City for Science and Technology, Riyadh, Saudi Arabia; 2 Center of Excellence for Genomics (CEG), King Abdulaziz City for Science and Technology, Riyadh, Saudi Arabia; 3 National Center for Stem Cell Technology, King Abdulaziz City for Science and Technology, Riyadh, Saudi Arabia; 4 Institute of Innovation and Industrial Development, King Abdulaziz City for Science and Technology, Riyadh, Saudi Arabia; 5 National Center for Biotechnology, King Abdulaziz City for Science and Technology, Riyadh, Saudi Arabia; University of Alabama at Birmingham, UNITED STATES

## Abstract

Small heat shock protein beta-1 (*HSPB-1*) plays an essential role in the protection of cells against environmental stress.Elucidation of its molecular, structural, and biological characteristics in a naturally wild-type model is essential. Although the sequence information of the *HSPB-1* gene is available for many mammalian species, the *HSPB-1* gene of Arabian camel (Arabian camel *HSPB-1*) has not yet been structurally characterized. We cloned and functionally characterized a full-length of Arabian camel *HSPB-1* cDNA. It is 791 bp long, with a 5′-untranslated region (UTR) of 34 bp, a 3′-UTR of 151 bp with a poly(A) tail, and an open reading frame (ORF) of 606 bp encoding a protein of 201 amino acids (accession number: MF278354). The tissue-specific expression analysis of Arabian camel *HSPB-1* mRNA was examined using quantitative real-time PCR (qRT-PCR); which suggested that Arabian camel *HSPB-1* mRNA was constitutionally expressed in all examined tissues of Arabian camel, with the predominately level in the esophagus tissue. Peptide mass fingerprint-mass spectrometry (PMF-MS) analysis of the purified Arabian camel *HSPB-1* protein confirmed the identity of this protein. Phylogenetic analysis showed that the *HSPB-1* protein of Arabian camel is grouped together with those of Bactrian camel and Alpaca. Comparing the modelled 3D structure of Arabian camel *HSPB-1* protein with the available protein 3D structure of *HSPB-1* from human confirmed the presence of *α*-crystallin domain, and high similarities were noted between the two structures by using super secondary structure prediction.

## Introduction

The one-humped camel, *Camelus dromedarius* (also known as Arabian camel), is one of the most important member of the Camelidae family. Arabian camel has played a major role in the culture and way of life in the Arabian Peninsula over the past couple thousand of years [1]. This animal has acclimatized itself to live in the desert, and to survive under extreme environmental conditions by promoting the expression of several genes such as small heat shock genes, which encode a family of proteins known as small heat shock proteins *sHSPs* [2–6]. They play a crucial role in Arabian camel defense from adverse environmental conditions by protecting other proteins from irreversible aggregation [7].

Small heat shock protein beta-1 (*HSPB-1*), a typical member of the *sHSPs* family, is a ubiquitously conserved ATP-independent protein, which is immensely preserved in a wide spectrum of organisms, ranging from bacteria to eukarya [8, 9]. Although the diversification in structure and function is a characteristic of most members of the *HSP* families, including *HSP90* [10, 11], *HSP70* [12], and *HSP40* [13], remarkable diversity is noted in *sHSP* family ranging from a single homologue of *sHSP* in *Saccharomyces cerevisiae* to over 20 homologues of *sHSPs* in plants [14, 15]. However, ten well-known members of the *HSPB* family (*HSPB-1* to *HSPB-10*) have been well-studied in human and mammals [16, 17]. These proteins are molecular chaperones, which commonly have a low molecular weight ranging from 12 to 30 kDa, and are generally distinguished by the presence of a typically conserved *α*-crystallin domain (ACD) that is flanked by a less conserved C-terminal extension (CTE) and an N-terminal domain (NTD) [18–20]. The formation of a stable dimer interface between two contiguous monomers of small heat shock proteins’ ACD facilitate the assembly of a large oligomers’ subunits [21, 22]. These molecular oligomers act as chaperones by binding to the unfolded proteins. Generally, the cellular concentration of many *sHSPs* is considerably increased in response to various of stresses, but they can also function fundamentally in many organisms and tissues [23].

Although *HSPB-1* protein is highly conserved across species from bacteria to mammals, *HSPB-1* protein from Arabian camel has not yet been characterized. This study aimed to clone and sequence a full-length of Arabian camel *HSPB-1* cDNA and determine amino acid sequence as well as elucidate its protein structure. In addition, we investigated the Arabian camel *HSPB-1* mRNA expression profile in ten different tissues. We believe that the study of biochemical and biophysical aspects of Arabian camel *HSPB-1* gene is likely to provide molecular insights into Arabian camel physiology as well as providing annotation of Arabian camel *HSPB-1* protein on which to advance further studies of Arabian camel proteins.

## Materials and methods

### Sample collection

Ten different Arabian camel tissue samples, including brain, lung, liver, kidney, testis, spleen, heart, stomach, skin, and esophagus, were obtained from male Arabian camel slaughtered at the main slaughter-house located in Saudi Arabia, Riyadh. This slaughter house is officially supervised by Veterinaries. Tissue samples to be used for RNA analysis were instantly immersed in RNAlater^®^ RNA Stabilization reagent (Qiagen, Ambion, Inc, USA) to prevent RNA degradation. The samples were then stored at -80°C until further use. While those other sample tissues to be used for protein analysis were transported on ice to the laboratory.

### Cell culture

Arabian camel skin fibroblast cell line (SACAS) was kindly provided by A. Alawad and routinely maintained as previously described [24]. Cells were used after they reached ≃ 70% confluency. Control cells were incubated at 37°C and experimental cell culture plates were incubated at 42°C for heat stress studies in 5% CO_2_ incubator at different time points 2, 4, 6, and 8 h. At each time point, cells were washed twice with cold PBS and lysed for RNA extraction using TRIZOL^®^ Reagent [25].

### Total RNA isolation and cDNA synthesis from tissues

Samples of 50 mg of each preserved tissues were subjected for RNA isolation. The tissues were homogenized in RTL lysis based buffer (Qiagen) containing 1% 2-mercaptoethanol by using steel beads (Sigma) and Tissue Lyser ⨿ (Qiagen). Nanodrop spectrophotometer (NanoDrop, ThermoScientific) was used to quantify samples at 260nm and the quality of RNA samples was evaluated using denaturing SYBR safe agarose gel 1% electrophoresis. Next, ≃ 2μg of total RNA were transcribed to first-stranded cDNA by using an ImProm-⨿ Reverse Transcription System (Promega, USA).

### Examining gene expression by using PCR and qRT-PCR

Gene-specific primers (Table 1) were designed based on the data from the Arabian camel genome project (http://camel.genomics.org.cn/page/camel/index.jsp). The PCR reaction mixture was carried out in a final volume of 25 μl, containing 12.5 μl 2X GoTaq^®^ Green Master Mix(Promega, USA), 1 μl of 5 pmol of each primer, 2 μl of cDNA. The PCR condition was 1 cycle at 94°C for 5 min, followed by 30 cycles at 94°C for 5 sec, 60°C for 30 sce, and 72°C for 45 sec. The final extension step was performed at 72°C for 10 min. The PCR products were then examined on 1.2% agarose gel stained with SYBR safe. In addition, the level of relative expression of Arabian camel *HSPB-1* mRNA was evaluated by examining the ten different Arabian camel tissues by using fluorescent quantitative real-time PCR (qRT-PCR) detector ViiA 7 Real-Time PCR System. The *β*-actin mRNA was used as a house keeping gene control. In this experiment, Fast SYBR^®^ Green Master Mix kit was used, and gene-specific primer pairs were designed to amplify 83 bp length of Arabian camel *HSPB-1*. The qRT-PCR reaction mixture were 10 μl of Fast SYBR^®^ Green Master Mix (Cat. No., 4385612, Applied Biosystems), 1 μl of the forward primer, 1 μl of the reverse primer, 3 μl of nuclease-free water and 5 μl of cDNA target, in a total volume of 20 μl. Thermal cycling parameters were initial denaturation at 95°C for 3 min, amplification of 40 cycles at 95°C for 3 s, and 60°C for 40 s.

**Table 1:** Gene-specific primers: The underlined bases represent the restriction sites used for cloning.

Usage	Primer name	Primer sequence 5′ → 3′	Product length (bp)
ORF-PCR	Camel HSPB1-F	GAGCCACCATGGCCGAG	759
	Camel HSPB1-R	GCCGGCAGGAACTTAGAACT	
	Camel HSPB1-pF	CACGGATCCGAGCCACCATGGCCGAG	
	Camel HSPB1-pR	CACGCGGCCGCTCAGCCGGCAGGAACTTAGAACT	
	*β*-Actin-F	CCCATTGAGCATGGCATCGT	291
	*β*-Actin-R	GTAGATGGGCACAGTGTGAG	
cDNA-RACE	cDNA-RACE-5’	CTTGGTCTTGACCGTCAGCTCCTC	283
	cDNA-RACE-3’	AGATCACCATCCCTGTCACCTTCGA	382
qRT-PCR	Camel HSPB1-qF	GTGTCGGAGATCCAGCAGAC	83
	Camel HSPB1-qR	TTCGTGCTTGCCAGTGATCT	
	*β*-Actin-qF	CCCATTGAGCATGGCATCGT	190
	*β*-Actin-qR	GTAGATGGGCACAGTGTGAG	

### Cloning and sequencing of Arabian camel *HSPB-1* cDNA

Rapid amplification of cDNA ends (RACE) was used to identify and isolate the 5′- and 3′-end of Arabian camel *HSPB-1* by using a RACE kits (Invitrogen, Carlsbad, CA, USA). Total RNA was annealed with 5′- and 3′-end primers (Table 1), and reversely transcribed respectively to the respective 5′- and 3′-cDNA. The resulting first-stranded 5′- and 3′-cDNA were then utilized as templates in PCR. The cycling program was set for five cycles of 95°C for 4 min; 5 cycles of 95°C for 15 s, 70°C for 15 s, 72°C for 3 min; 5 cycles of 95°C for 15 s, 68°C for 15 s, 72°C for 3 min; 5 cycles of 95°C for 15 s, 65°C for 15 s, 72°C for 3 min; 25 cycles of 95°C for 15 s, 60°C for 15 s, 72°C for 3 min; 1 cycle of 72°C for 5 min. The purified nested PCR product was ligated into pcDNA5/FRT/TO GFP-tagged vector (a gift from Harm Kampinga; Addgene plasmid No., 19487) [26] by using BamHI (NEB R3136S) and NotI (NEB R3189S) restriction sites. Subsequently, 5 ul of the ligation mixture was used as a template to transform chemically modified DH5*α* competent cells (ThermoFisher Scientific). The cloned Arabian camel *HSPB-1* was sequenced using Applied Biosystems 3730xl DNA Analyzer platform (Applied Biosystems, Foster City, USA). The conditions of the chain termination PCR were as follows: one cycle at 94°C for 35 s, followed by 25 cycles at 94°C for 40 s, 50°C for 35 s, and 60°C for 1 min.

### Protein extraction and quantification

Proteins from brain, testis, kidney, liver, lung, and the spleen were extracted using RIPA lysis buffer. Next, 4 mg sample from each tissue was homogenized in 4 mL of RIPA lysis buffer containing 5 M NaCl, 0.5 M EDTA, 1 M Tris-HCl, NP-40, 10% soudium deoxycholate, and 10% SDS by using steel beads (Sigma) and a Tissue Lyser ⨿ (Qiagen). Lysates were then centrifuged at 14,000 rpm for 1 hour at 4°C. The supernatant fractions were then collected, and total protein quantity for each tissue was determined using the bicinchoninic acid assay (BCA).

### Arabian camel *HSPB-1* protein identification by using LC-MS

For this, 25 μg of Arabian camel protein lysates were subjected to one-dimensional sodium dodecyl sulfate polyacrylamide gel electrophoresis (1-D SDS-PAGE) by using 4% staking and 15% resolving polyacrylamide gels (1 mm thickness gel) by running for 120 min. The 1-D SDS-PAGE was then stained overnight in a solution containing the mixture of Coomassie R-240, 40% methanol, and 10% acetic acid. The gel was subsequently destined in a solution containing 30% methanol and 10% acetic acid.

The excised band gel piece holding proteins with molecular weight of approximately 20-25 kDa was cut into cubes and incubated for 45 min in 300 μl of 1:1 mixture of 100 mM ammonium bicarbonate buffer containing 50% acetonitrile and was vortexed for 10 min; the supernatant was then discarded. The procedure was repeated until the stain was completely removed. Next, 10 mM dithiothreitol (DTT) in 100 mM ammonium bicarbonate buffer was added to the gel cubes in order to reduce the disulfide bonds; the cubes were incubated for 30 min at 56°C in an air thermostat. After they were rinsed in 100 μl of acetonitrile, 200 μl of 50 mM iodoacetamide solution was added, and the mixture was incubated for 20 min at room temperature in the dark.

The gel cubes were then dehydrated twice with 100% acetonitrile for 10 min each and then dried in a speed-vac for 10 min in order to process them ready for tryptic digestion. Trypsin (10ng/ul) solution was added to the dried gel cubes just enough to cover the gel cubes and incubated for 10 min at room temperature. Subsequently, 100 mM ammonium bicarbonate buffer was added until the gel cubes were immersed which was then incubated at 37°C for overnight. The digestion was then stopped by adding 20 μl of 5% formic acids. The digested solution (extracted peptides) was then transferred to a clean autosampler vial.

Millipore^®^Ziptips C18 pipette (Tip size:P10, Merck KGaA, Darmstadt, Germany) was used to prepare sample for Matrix-assisted laser desorption/ionization-time of flight (MALDI-TOF) mass spectrometry. The Ziptip pipette was washed with 100% methanol, followed by 0.1% trifluroacetic acid (TFA) solution. The tryptic-cleaved peptides mixture were then loaded onto the Ziptip pipette and then were desalted using 0.1% TFA. The loaded peptides were then eluted in 10 μl of *β*-cyano-4-hydroxycinnamic acid, which was used as a matrix. 1 μl of aliquots were generally sampled directly from the digest supernatant for MS fingerprint analysis by using Axima Performance ^®^ MALDI TOF/TOF Mass Spectrometer (Shimadzu Corporation, UK). The data were searched using the MASCOT search engine (http://www.matrixscience.com).

### Phylogenetic tree

The Arabian camel *HSPB-1* protein sequence was used as a query to retrieve 40 *sHSP* sequences from the NCBI Protein Database. The *α*-crystalline domain was verified in all the retrieved protein sequences by using InterProScan [27] at (https://www.ebi.ac.uk/interpro/) (S3 Table). To verify whether the Arabian camel *HSPB-1* protein is distinctly related to the *HSPB-1* proteins family, we retrieved 40 *HSP* orthologues, which are conspicuously related to ten well-known *sHSP* families known as *HSPB-1* to *HSPB-10*. To ensure the consistency of sampling, we retrieved all *sHSPs* proteins orthologues from the same species. We used Arabian camel *HSPB-1* protein sequence as a query to search the NCBI Protein Database to identifying *HSPB-1* proteins across diverse vertebrate species. Another set of *sHSP* members were sampled from the same mammalian species to ensure the consistency of sampling. The accession numbers of protein members investigated are listed in (S3 Table). Consequently, the full length amino acid sequences, including Arabian camel *HSPB-1* protein, were selected for multiple alignment by using CLUSTALX 2.1 program [28]. A bootstrap re-sampling technique was used to ensure the robustness of the generated topological tree. Neighbor Joining (NJ) phylogenetic analysis was conducted in MEGA 7.0 [29]. The constructed topological trees were depicted and edited using FigTree v1.4.3. (http://tree.bio.ed.ac.uk/software/figtree/).

### Structure modeling

The secondary structure of Arabian camel *HSPB-1* protein sequence was generated using Geneious software v10.0.3 [30]. Consequently, a three-dimensional (3D) structure of Arabian camel *HSPB-1* protein containing 201 residues was predicted after submitting the protein sequence to Phyre2 server (http://www.sbg.bio.ic.ac.uk/phyre2/html/page.cgi?id=index). The similarities between modeled Arabian camel *HSPB-1* and human *HSPB-1* structure (PDB:2YGD) were superimposed by using Pymol software. The quality of the superimposed 3D structures was assessed using PDBe on (https://swissmodel.expasy.org/interactive). The antigenicity, hydrophobicity, and flexibility of Arabian camel *HSPB-1* protein were predicted according to the methods of Kolaskar, Parker, and Karplus, respectively [31, 32].

## Results

### Tissue-specific expression profile of Arabian camel *HSPB-1* mRNA

**Fig 1:**
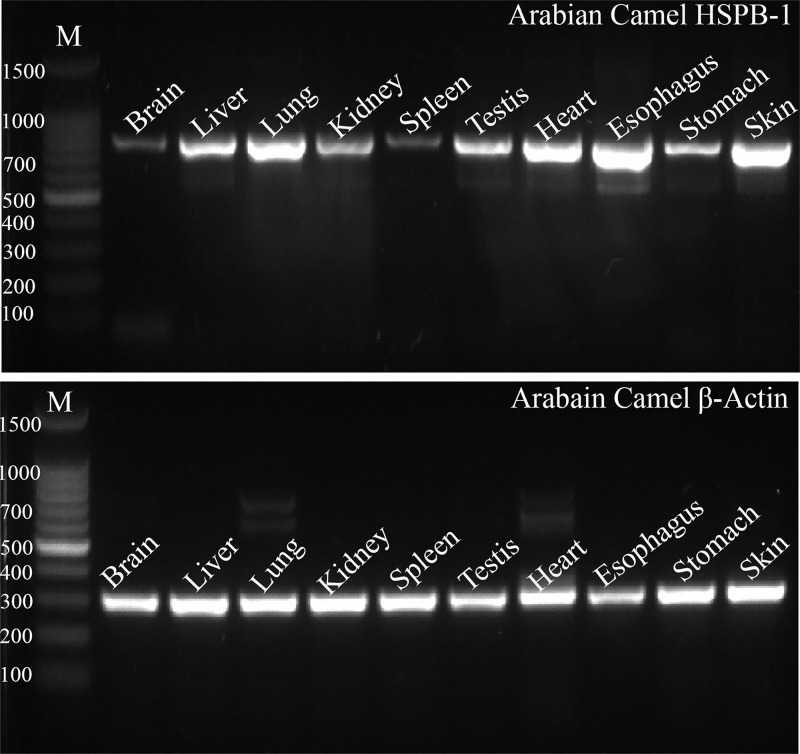
Agarose gel (1.2%) electrophoresis of PCR products for *HSPB-1* and *β*-actin Arabian camel mRNAs, 1500 bp DNA molecular weight marker was used.

**Fig 2:**
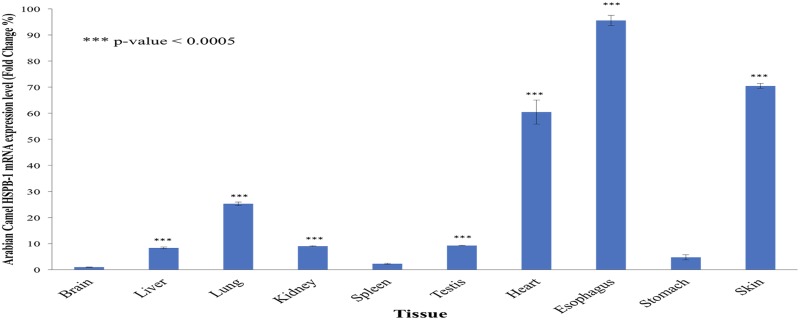
Arabian camel *HSPB-1* mRNA expression levels in different tissues. The results are expressed relative to that of *β*-actin as an endogenous control.

The expression of Arabian camel *HSPB-1* mRNA was found in all examined tissues of Arabian camel (Fig 1), indicating its important role in cellular proteostasis. Specific primers (Table 1) were designed to amplify a single 759 bp for Arabian camel *HSPB-1* and 291 bp for Arabian camel *β*-actin genes (as endogenous control). In addition, the level of expression of Arabian camel *HSPB-1* mRNA in the ten different tissues was studied using qRT-PCR. The qRT-PCR primers were designed to amplify 83 and 190 bp for Arabian camel *HSPB-1* and *β*-actin, respectively. Under no heat stress condition, the maximum expression of Arabian camel *HSPB-1* mRNA was noted in the Arabian camel esophagus, skin, and heart, followed by nearly equally expression in the liver, kidney, testis, and lung, whereas the lowest expression was noted in the brain, spleen, and stomach tissues (Fig 2). This result is in agreement with that of a previous study investigating tissue-specific expression of *HSPs* in buffalo tissues [33]. These observations might be significant in understanding the differential sensitivities of Arabian camel tissues to environmental conditions.

**Fig 3:**
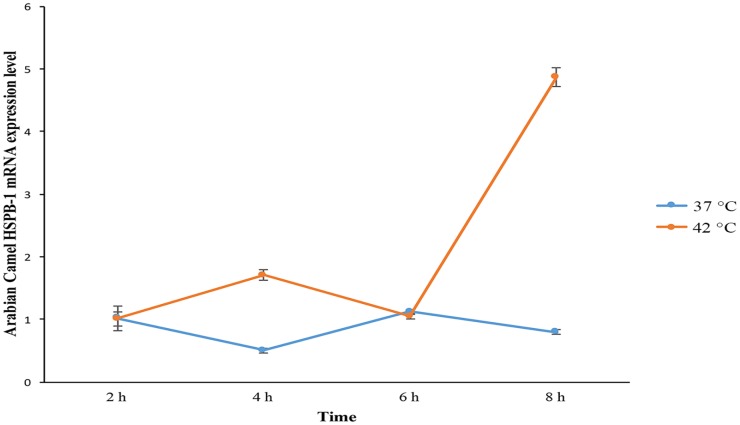
Arabian camel *HSPB-1* mRNA expression levels in SACAS cells at control(37°C) and heat-stressed condition (42°C) for different time points. The results are expressed relative to that of *β*-actin as an endogenous control.

In order to investigate the effect of heat stress(42°C) on the level of expression of Arabian camel *HSPB-1* mRNA, we used SACAS cells as a model system by using qRT-PCR. The camel skin fibroblasts were exposed to elevated ambient temperature (42°C) at different time points. The expression of *HSPB-1* mRNA, as shown in (Fig 3), was remarkably upregulated in response to the 42°C heat stress after 6h incubation compared with that in the control at 37°C. This result showed that the induction of Arabian camel *HSPB-1* mRNA expression depended on the duration and temperature of heat stress.

### Characterization and sequence of the full-length of *HSPB-1* cDNA from Arabian camel

**Fig 4:**
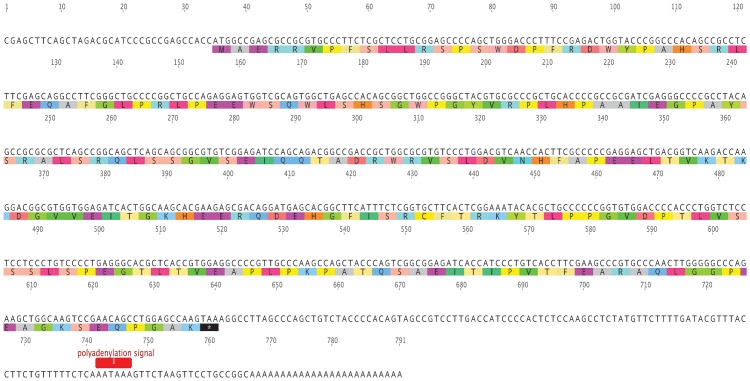
Nucleotide and amino acid sequences of Arabian camel *HSPB-1* cDNA (GenBank accession no., MF278354). The numbers above the nucleotide sequence show the nucleotide positions. The stop codon is represented with an asterisk(*). The putative polyadenylation signal is shown in red.

The full-length of Arabian camel *HSPB-1* cDNA contained a 5′-untranslated region (UTR) of 34 bp, a 3′-UTR of 151 bp with typical polyadenylation signal (AATAAA), and with a poly(A) tail were obtained and deposited as GenBank accession No. MF278354. The open reading frame (ORF) includes 606 bp and encodes a protein of 201 residues (Fig 4). The sequence indicated a length of 791 bp, and revealed high statically significant similarity scores to many *HSPB-1* nucleotide sequences from other species. The Bactrian camel (*Camelus bactrianus*) showed the highest homology score of 99%, followed by that of alpacas (*Vicugna pacos*); suggesting a close evolutionary relationship. The other mammals shared a high identity score ranging from 82% to 92%, as shown in (Table 2).

**Table 2:** Nucleotide homology of Arabian camel *HSPB-1* with that from other species.

Species	Common name	Accession no.	length (bp)	Identity (%)
*Homo sapiens*	Human	BC000510.2	867	85
*Pan troglodytes*	Chimpanzee	XM_519162.5	947	85
*Mus musculus*	Mouse	NM_013560.2	913	82
*Sus scrofa*	Pig	NM_001007518.1	624	91
*Equus caballus*	Horse	XM_001504478.3	923	88
*Bos taurus*	Cattle	AB605262.1	672	92
*Camelus dromedarius*	Arabian Camel	MF278354	791	100
*Capra hircus*	Goat	XM_018040903.1	891	91
*Camelus bactrianus*	Bactrian Camel	XM_010972325.1	849	99
*Vicugna pacos*	Alpaca	XM_015236804.1	820	99
*Macaca mulatta*	Rhesus monkey	NM_001260949.2	893	85
*Ailuropoda melanoleuca*	Giant panda	NM_001304892.1	633	90
*Canis lupus familiaris*	Dog	NM_001003295.2	864	87
*Macaca fascicularis*	Crab-eating macaque	NM_001283885.1	845	85

### Identification of Arabian camel *HSPB-1* protein by using mass spectrometry

**Fig 5:**
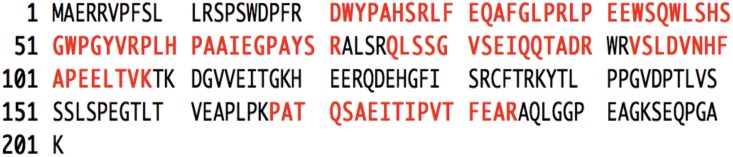
MLDI-TOF MS-derived peptides (red) matched to the sequence of Arabian camel *HSPB-1* protein(accession no. ATJ03466).

For peptide mass fingerprint mass spectrometry (PMF-MS), the targeted protein band (spot) was manually excised from the gel (S1 Fig) and was subjected to MS analysis. Of the total trypsin-digested peptide mass of Arabian camel *HSPB-1* protein, seven peptides, which covered 49% of the entire protein sequence, were hit in NCBIprot database (containing 4114420 sequences) by using the Mascot peptide fingerprint search engine with Arabian camel *HSPB-1* protein (accession no. ATJ03466) with a score of 125 and p < 0.05 (Fig 5).

The mass spectrum revealed several protonated ions [M+H]+ in the peptide fragments. As listed in (Table 3), the ions at 1031.89, 1178.04, 2314.72, 1479.23, 1619.27, 1798.42, and 1831.55 were the seven trypsin digested peptides corresponding to amino acids 21-28, 29-38, 39-57, 58-71, 76-90, 93-108, and 168-184, respectively. As interpreted in (Table 3), the peptide mass profiles were obtained from NCBIprot database search engine, and amino acid sequence of individual peptides were identified from the sequence of Arabian camel *HSPB-1* protein from the desired spot of this protein on the SDS-PAGE. The PMF-MS results were also homologous with that in some other animals; the second best matching protein received a score of 102 for Alpaca (accession no. XP_015092290) *HSPB-1* protein. The third and fourth best matching proteins were scored with 100 and 80 for Bactrianus camel (accession no. XP_010970627) and pig (accession no. NP_001007519.1) HSPB-1 proteins, respectively.

**Table 3:** Calculated and observed ions of peptide masses of Arabian camel *HSPB-1* protein.

Amino Acid positions	[M+H]+		
Start-End	Observed(m/z)	Calculated(m/z)	Peptide Sequence
21-28	1031.89	1030.46	DWYPAHSR
29-38	1178.04	1176.63	LFEQAFGLPR
39-57	2314.72	2313.1	LPEEWSQWLSHSGWPGYVR
58-71	1479.23	1477.77	PLHPAAIEGPAYSR
76-90	1619.27	1617.8	QLSSGVSEIQQTADR
93-108	1798.42	1796.93	VSLDVNHFAPEELTVK
168-184	1831.55	1829.95	PATQSAEITIPVTFEAR

### Characterization of *HSPB-1* protein from Arabian camel

**Fig 6:**
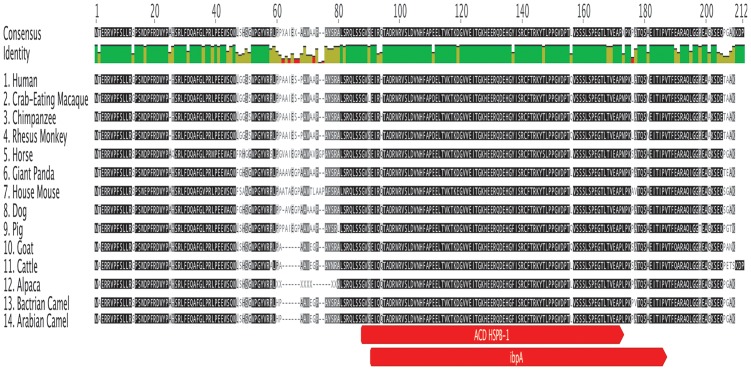
Multiple alignment of amino acid sequence of Arabian camel *HSPB-1* protein with that in other 13 mammalian species. Identical amino acids are marked in green color, and typical ACD and ipbA domains are showed in red.

The protein sequence was compared with those of other mammalian *HSPB-1* protein sequences by using ClustalW alignment [34], as shown in (Fig 6). Results of multiple sequence alignment of Arabian camel *HSPB-1* protein showed two highly conserved domains of about 85 residues (from 88 to 173): ACD and IbpA domains, which were flanked by less conserved NTD and CTE across the species. The comparative analysis of Arabian camel *HSPB-1* protein sequence showed high similarity with that of other vertebrates (Table 4). As expected, the highest homology was observed between Arabian camel *HSPB-1* protein and the one from Bactrian camel (99%). The other vertebrates showed a high homology ranging from 86% to 95%, as shown in (Table 4). The complete amino acid sequence of Arabian camel *HSPB-1* is shown in (Fig 5).

**Table 4:** Amino acids homology of Arabian camel *HSPB-1* with that in other species.

Species	Common name	Protein (Accession no.)	Protein length	Identity (%)
*Homo sapiens*	Human	NP_001531	205	86
*Pan troglodytes*	Chimpanzee	XP_519162.3	205	86
*Mus musculus*	Mouse	NP_038588	209	86
*Sus scrofa*	Pig	NP_001007519	207	92
*Equus caballus*	Horse	XP_001504528	209	87
*Bos taurus*	Cattle	NP_001020740	204	95
*Camelus dromedarius*	Arabian Camel	ATJ03466	201	100
*Capra hircus*	Goat	XP_017896392	201	95
*Camelus bactrianus*	Bactrian Camel	XP_010970627	201	99
*Vicugna pacos*	Alpaca	XP_015092290	197	94
*Macaca mulatta*	Rhesus monkey	NP_001247878.1	205	86
*Ailuropoda melanoleuca*	Giant panda	NP_001291821.1	207	90
*Canis lupus familiaris*	Dog	NP_001003295.2	206	89
*Macaca fascicularis*	Crab-eating macaque	NP_001270814.1	205	86

Considering the amino acid composition, the average isoelectric point (pI) for Arabian camel *HSPB-1* protein calculated using a computer algorithm [35] was found to be 6.162 (S2 Fig), and its estimated molecular weight was 22.382 kDa. The basic, acidic, charged, polar, and hydrophobic amino acids were 22 (10.95%), 25 (12.44%), 58 (28.86%), 47 (23.38%) and 65 (32.34%), respectively. The hydrophobic and aromatic amino acids are overrepresented in the NTD, whereas polar and charged ones are underrepresented [36]. The instability of Arabian camel *HSPB-1* protein was calculated to be 64.99, and hence this protein was classified as unstable. The molar extinction coefficient was found to be 39085±5% *cm*^−^1 *M*^−^1. The amino acids composition of Arabian camel *HSPB-1* protein is shown in (S1 Table).

**Fig 7:**
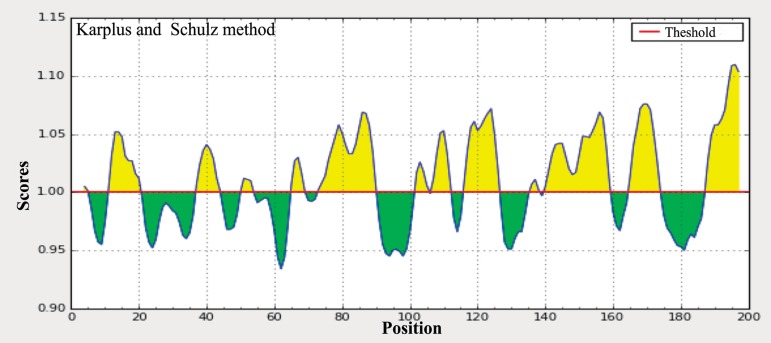
Karplus and Schulz flexibility prediction of Arabian camel HSPB-1 protein. The x-axis and y-axis represent the position and score, respectively. The threshold is 1.0. The flexible regions of the protein are shown in yellow color, above the threshold value.

**Fig 8:**
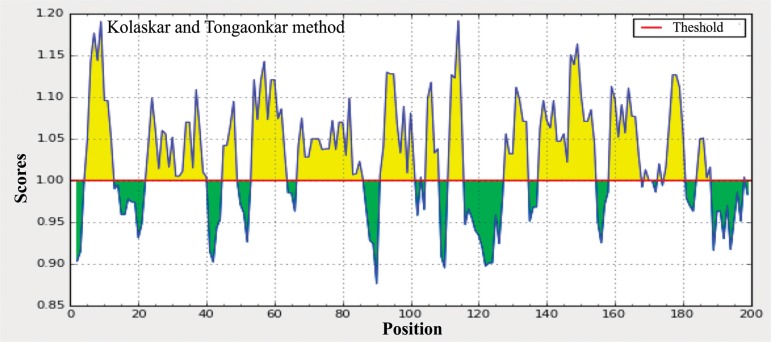
Kolashkar and Tongaonkar antigenicity prediction of the most antigenic regions of Arabian camel *HSPB-1* protein. The threshold value is 1.0. The regions above the threshold are antigenic, shown in yellow.

The protein structural flexibility was predicted from the amino acid sequence of Arabian camel *HSPB-1* protein by using the Karplus and Schulz method [32], in which the size of window was optimized to 7 residues (Fig 7). The flexcibility analysis showed that Arabian camel *HSPB-1* protein was more flexible at its C-terminal than at the N-terminal regions, and thus possibly also the surface amino acids in this protein might be considered as epitopes. In addition, Arabian camel *HSPB-1* protein sequence was used as a query to identify B cell epitopes by using the Kolaskar and Tongaonkar antigenicity method [31] (Fig 8). The results showed that the average antigenic tendency value was 1.027 for the protein, with the minimum value of 0.876 and maximum of 1.192. This protein harbors nine antigenic peptides, the lengths of which range from 6 to 20 amino acids (S2 Table). The results also revealed that the two regions from 8 to 11 and 113 to 116 amino acids were the most preferred B cell epitope characteristics.

**Fig 9:**
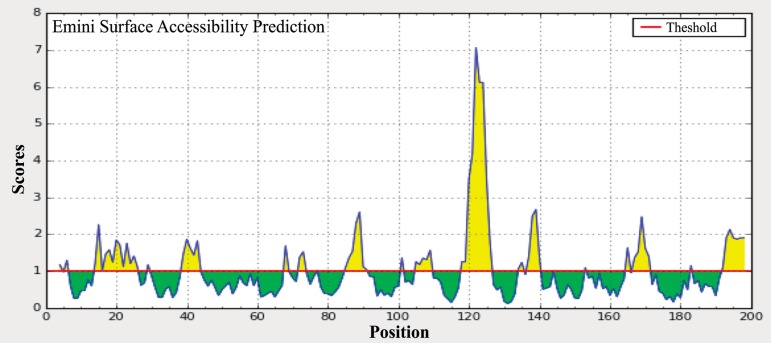
Emini surface accessibility prediction of Arabian camel *HSPB-1* protein. The threshold value is 1.000. The regions above the threshold are antigenic and are shown in yellow.

**Fig 10:**
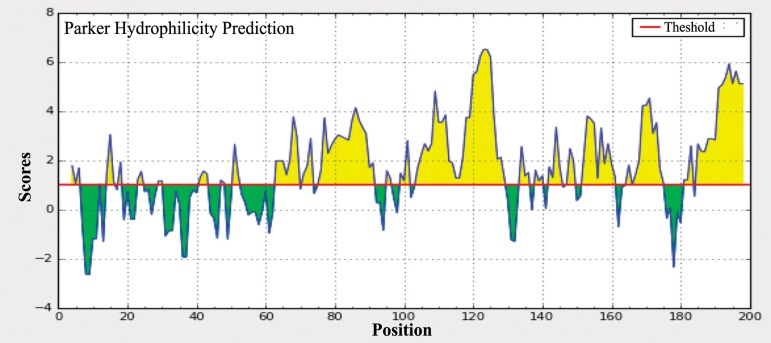
Parker hydrophilicity prediction of Arabian camel *HSPB-1* protein. The threshold is 1.0. The regions having *β*-turns in the protein are shown in yellow color, above the threshold value.

**Fig 11:**
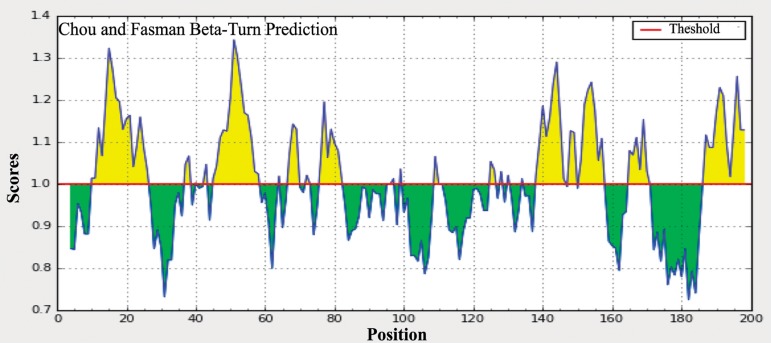
Chou and Fasman *β*-turns prediction of Arabian camel *HSPB-1* protein. The threshold is 1.00. The regions having *β*-turns in the protein are shown in yellow color.

The amino acid sites that are located on the surface of Arabian camel *HSPB-1* protein were predicted using the Parker hydrophilicity tool [37] and the Emini surface accessibility prediction [38]. Those sites might increase the probability of predicting the antigenic regions since they are more accessible and hydrophilic than the interior regions of the protein. The maximum surface probability value was found to be 5.823 from amino acid position 121 to 126 for Arabian camel *HSPB-1* protein (Figs 9 and 10). In addition, *β*-turns structure in a protein are mostly hydrophilic and surface accessible in nature. The *β*-turns were also predicted in Arabian camel *HSPB-1* protein by using Chou and Fasman Beta turn prediction [39]. The results suggested that this protein is rich in *β*-turns in the region between 80 to 175 residues, which is the region where *β*-strands are oriented in anti-parallel to form *β*-sheets (Fig 11).

**Fig 12:**
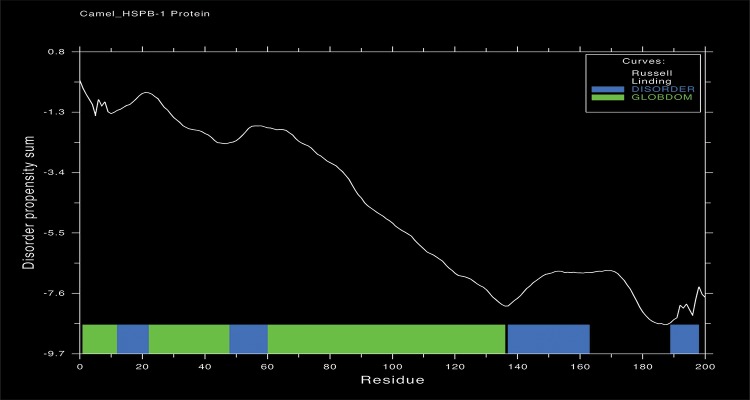
Glob Plot analysis. Blue boxes are disordered regions, and green boxes are ordered regions in the Arabian camel *HSPB-1* protein.

GlobPlot server (http://globplot.embl.deis) was used in order to predict the disordered and ordered (globular) regions within Arabian camel *HSPB-1* protein. In this program, ordered regions are described as those have regular secondary structure (*α*-helices and *β*-strands), whereas disordered ones are that lack such structures. The Russell/Linding [40] set was selected, in which *α*-helices and *β*-strands structures are assigned as globular regions (GlobDoms), whereas random coils and *β*-turns structures as disordered regions. Residue ranges for the disordered regions (blue) and globular regions (green) are shown at the bottom of the graph (Fig 12).

### Phylogeny and classification of Arabian camel *HSPB-1* protein

**Fig 13:**
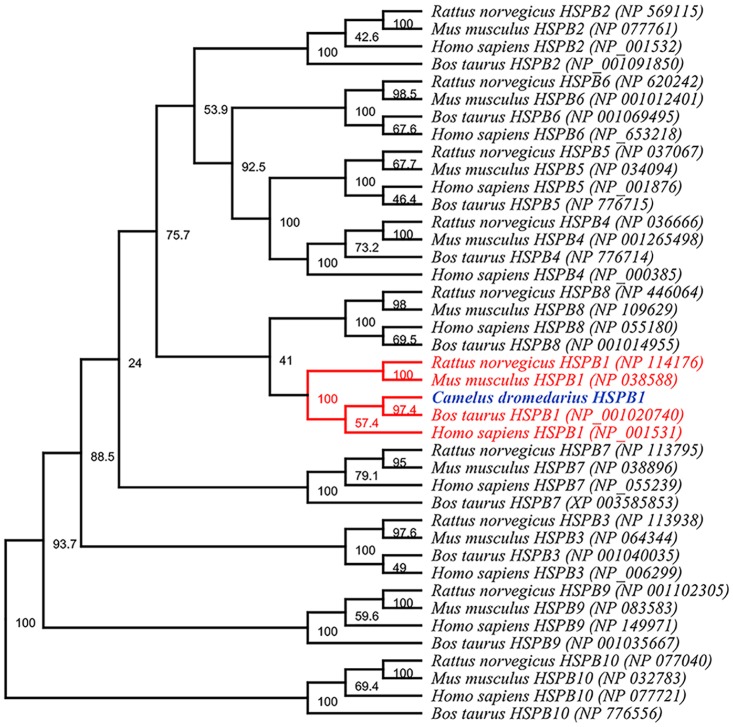
Phylogenetic tree shows the classification of Arabian camel *HSPB-1* within the sHSPB family.

**Fig 14:**
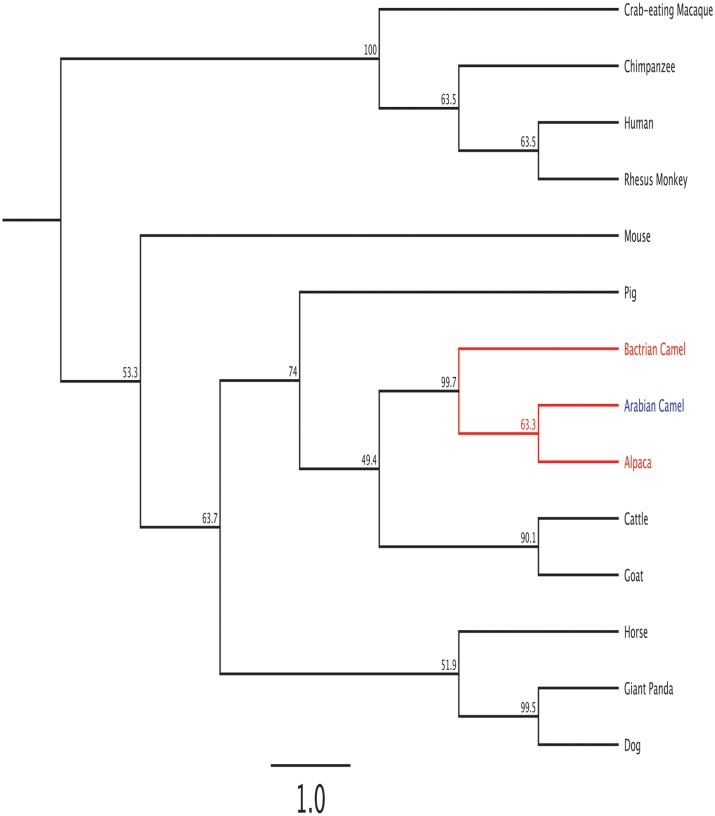
The phylogenetic tree shows the relationship of camel *HSPB-1* protein and protein sequences from other species. Maximum likelihood tree based on complete coding sequences. Values at nodes are bootstrapping ≥ 49% obtained from 1000 resampling of the data.

After confirming the relationship of Arabian camel *HSPB-1* protein to the *HSPB-1* family, we constructed phylogenetic trees by using the Arabian camel *HSPB-1* protein sequence as a query to retrieve 40 orthologues sequences derived from different vertebrate species (S3 Table). the NJ phylogenetic trees were constructed based on the multiple alignment of the *HSPB-1* protein sequences (Fig 13). The depicted topology showed that the Arabian camel *HSPB-1* clustered closely with even-toed ungulates’ *HSPB-1* into two distinct clades. In addition, the evolutionary position of Arabian camel was shown in a phylogenetic tree (Fig 14). The Arabian camel *HSPB-1* was grouped more closely with the Bactrian camel, alpaca from cattle,goat and further related with pig.

### Secondary and 3D structures of Arabian camel *HSPB-1* protein

**Fig 15:**
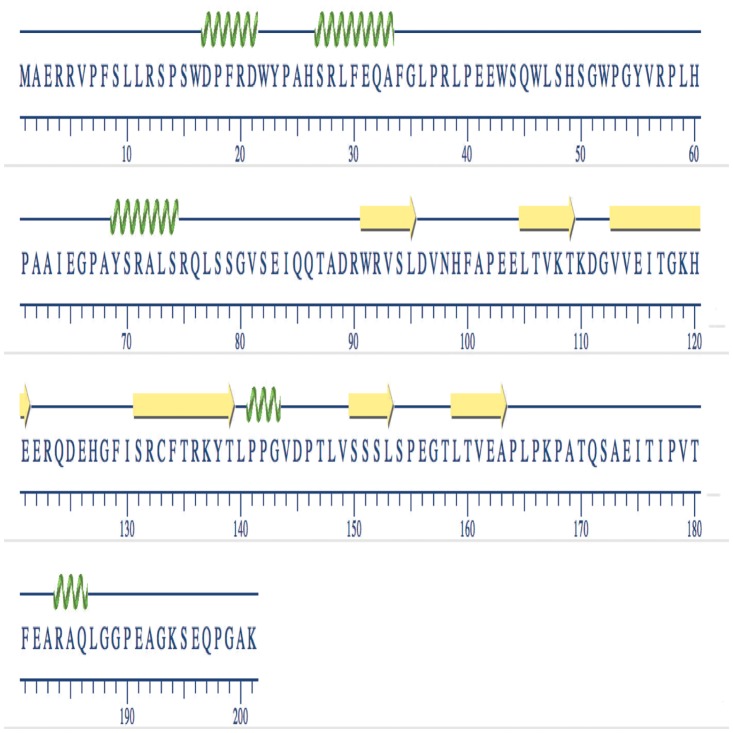
The secondary structure of Arabian camel *HSPB-1* protein.

**Fig 16:**
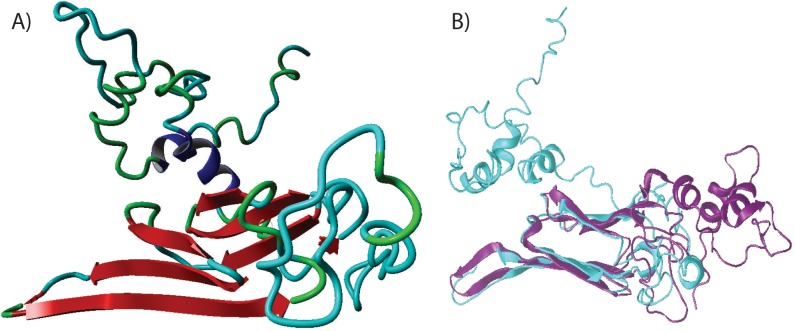
Modeled 3D structures. **(A)** The 3D structure of Arabian Camel *HSPB-1* protein. **(B)** Stereo ribbon representation of the predicted 3D structure model of Arabian camel *HSPB-1* (cyan) and the superimposition with *Homo sapiens* *α*-B-crystallin chain V (purple).

The primary structure of Arabian camel *HSPB-1* protein was used to predict its secondary structure, which shows the first level of protein folding. The predicted structure suggested that Arabian camel *HSPB-1* protein composed of 5 *α*-helices and 6 *β*-strands (Fig 15) in which the 6 *β*-strands forms a highly conserved ACD of approximately 85 residues (from 88 to 173), which is flanked by less conserved NTD and CTE. The 3D structure of this domain forms an immunoglobulin-like *β*-sandwich fold in the C-terminal half of the Arabian camel *HSPB-1* protein (Fig 16A). The ACD domain mediates the formation Arabian camel *HSPB-1* dimers via the anti-parallel pairing of the same *β*-strand from two monomers.

To construct the 3D structural model of Arabian camel *HSPB-1* protein, we generated its homology model by using Phyre2 server (http://www.sbg.bio.ic.ac.uk/phyre2/html/page.cgi?id=index) (Fig 16A). In this study, we used *Homo sapiens* *α*-B-crystallin chain V (PDB ID: 2YGD) [41] as a template in which 86% of amino acid residues were modeled at > 90% confidence. The 3D structural model consisted of 5 *α*-helices and 6 *β*-strands. The ACD region is folded into a compact of 6-anti-parallel-strands forming two *β*-sheets. It has a very similar fold and topology as those from human. The structural similarity of Arabian camel *HSPB-1* with human *HSPB-1* was examined by superimposing their structures by using the Pymol program (https://www.pymol.org/) (Fig 16B). The root mean square deviation between Arabian camel *HSPB-1* and human *α*-B-crystallin chain V structures was 3.134. The Q-score is another crucial parameter to assess the similarity of the homologous structures; it represented that the quality of recognition and superimposition was 0.8435, indicating high structural identity. The Z- and P-scores of the 3D structure of Arabian camel *HSPB-1* and human *HSPB-1* were 44.6 and 65.9, respectively.

**Fig 17:**
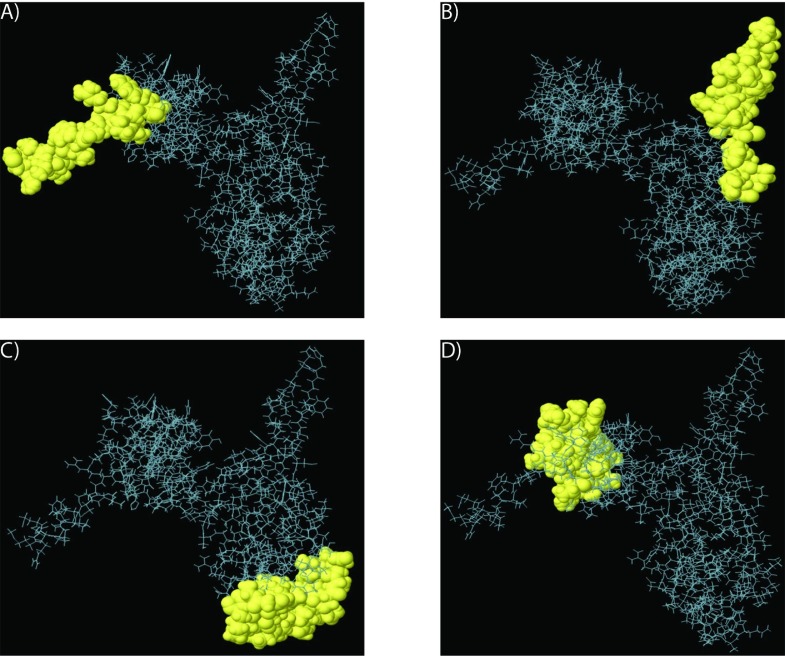
3D representation of discontinues epitopes (A to D) of Arabian camel *HSPB-1*. The epitopes are shown in yellow surface, and bulk of Arabian camel *HSPB-1* protein is shown in grey sticks.

The epitope regions of Arabian camel *HSPB-1* protein based on its 3D structure were predicted using Ellipro server (http://tools.iedb.org/ellipro/). Four discontinuous peptides were identified having score value of > 0.7. The highest probability of a discontinuous epitope was computed as 78.5%. Amino acids involved in discontinuous epitopes, their sequence location, number of amino acids, and scores are shown in (Table 5), whereas their positions on 3D structure of Arabian camel *HSPB-1* protein are shown in (Fig 17).

**Table 5:** Predicted discontinous antigenic epitopes of Arabian camel *HSPB-1* protein.

Start	End	Peptide	Peptide Length	Score	3D Structure
1	17	MAERRVPFSLLRSPSWD	17	0.785	A
119	137	KHEERQDEHGFISRCFTRK	19	0.753	B
166	201	PKPATQSAEITIPVTFEARAQLGGPEAGKSEQPGAK	36	0.72	C
33	58	AFGLPRLPEEWSQWLSHSGWPGYVRP	26	0.706	D

## Discussion

The sHSPs help maintain protein homeostasis by interplaying with unfolded substrates to prevent cellular damage [16]. The ATP-independent chaperone *HSPB-1* protein is a typical example. The *HSPB-1* protein is expressed in several tissue types under stress-induced conditions, where it serves as a chaperone for partly folded cellular proteins. Thus, a full structural description of Arabian camel *HSPB-1* gene is an important step toward understanding its mode of action.

The molecular characterization of Arabian camel *HSPB-1* gene is crucial for realizing the effect of exposure to different environmental factors on the health position of this animal. The study focused on the molecular characterization of sHSP family, mainly the HSPB-1 protein from *C. dromedarius*. We cloned Arabian camel *HSPB-1* cDNA(791 bp) by using specific primers spanning the entire ORF, encoding 201 amino acids for the protein with size of 22.382 kDa; highly matches with several *HSPB-1* protein sequences from other species were found (Table 2). Arabian camel *HSPB-1* cDNA sequence was matched with other 13 other mammalian *HSPB-1* sequences in GenBank and submitted in the NCBI database with the accession number MF278354.

Our findings suggest that Arabian camel *HSPB-1* mRNA is highly expressed in esophagus, skin, and heart, followed by nearly equally expressed in liver, kidney, testis, and lung, whereas the brain, spleen and stomach tissues showed the lowest levels of Arabian camel *HSPB-1* mRNAs, as indicated by qRT-PCR analysis (Fig 2). Heat-shock response can be determined by transcriptional activation of *HSP* genes and accumulation of their proteins [42, 43]. We utilized Arabian camel skin cell line as a model to investigate heat-stress responses. The effect of heat stress on the expression level of Arabian camel *HSPB-1* mRNA was examined by thermal stressing the cells at 42°C during an 8 h time course (Fig 3). As can be seen in (Fig 3), the expression level of Arabian camel *HSPB-1* mRNA increased after 6 h time course after 42°C heat stress, indicating that Arabian camel *HSPB-1* mRNA expression depends on time and temperature exposure. Further, heat-induced HSP27 expression was shown to mainly dependent on the time and temperature of exposure in skin and lung tissues of rats [44].

Two highly conserved domains are present in Arabian camel *HSPB-1* protein: ACD and IbpA domains that are localized near the C-terminal end of the protein. Multiple sequence alignment of Arabian camel *HSPB-1* protein (Fig 6) showed a highly conserved ACD of approximately 85 residues (from 88 to 173), which was flanked by less conserved NTD and CTE across the species. The NTD is very diverse in protein sequence and is therefore largely responsible for the sequence variation of *HSPB-1* protein between organisms. The amino acids such as Trp(W), Phe(F), and Pro(P) are overrepresented in the NTD. However, the CTE contains a highly conserved IXI motif, which is thought to be important for inter-dimer contacts [45].

In general, mammalian *HSPB-1* proteins exist as polydispersed oligomeric population, and their full crystal structures have not yet been determined. Nevertheless, the crystal structure of ACD of *HSPB-1* protein indicates a *β*-sheet rich immunoglobulin-like fold. The 3D structure of a protein provides useful information regarding its function. Comparative modeling is possible to predict the 3D structure of a protein based only on its primary structure. Therefore, we predicted the 3D structure of Arabian camel *HSPB-1* protein, of which the amino acid sequence is known. The 3D structure of Arabian camel ACD domain forms an immunoglobulin-like *β*-sandwich fold in the C-terminal half of the protein (Fig 16A). The ACD mediates the formation Arabian camel *HSPB-1* dimers via the anti-parallel pairing of the same *β*-strand from two monomers.

## Supporting information

S1 Fig SDS-PAGE (15%) followed by staining with the Coomassie blue, as indicated in the “Materials and methods” section. (EPS)

S2 Fig Isoelectric point (pI) of Arabian camel *HSPB-1* protein according to different scale calculation. (EPS)

S1 Table Chemical composition of Arabian camel *HSPB-1* protein. (XLSX)

S2 Table Kolaskar and Tongaonkar antigenicity analysis. (XLSX)

S3 Table The 40 orthologues sequences retrieved with Arabian camel protein. (XLSX)
